# Systematic review of influenza A(H1N1)pdm09 virus shedding: duration is affected by severity, but not age

**DOI:** 10.1111/irv.12216

**Published:** 2013-12-02

**Authors:** James E Fielding, Heath A Kelly, Geoffry N Mercer, Kathryn Glass

**Affiliations:** a Victorian Infectious Diseases Reference LaboratoryNorth Melbourne, Vic., Australia; b National Centre for Epidemiology and Population Health, The Australian National UniversityCanberra, ACT, 0200, Australia

**Keywords:** adult, antiviral agents, child, influenza A virus, H1N1 subtype, influenza, human, virus shedding

## Abstract

Duration of viral shedding following infection is an important determinant of disease transmission, informing both control policies and disease modelling. We undertook a systematic literature review of the duration of influenza A(H1N1)pdm09 virus shedding to examine the effects of age, severity of illness and receipt of antiviral treatment. Studies were identified by searching the PubMed database using the keywords ‘H1N1’, ‘pandemic’, ‘pandemics’, ‘shed’ and ‘shedding’. Any study of humans with an outcome measure of viral shedding was eligible for inclusion in the review. Comparisons by age, degree of severity and antiviral treatment were made with forest plots. The search returned 214 articles of which 22 were eligible for the review. Significant statistical heterogeneity between studies precluded meta-analysis. The mean duration of viral shedding generally increased with severity of clinical presentation, but we found no evidence of longer shedding duration of influenza A(H1N1)pdm09 among children compared with adults. Shorter viral shedding duration was observed when oseltamivir treatment was administered within 48 hours of illness onset. Considerable differences in the design and analysis of viral shedding studies limit their comparison and highlight the need for a standardised approach. These insights have implications not only for pandemic planning, but also for informing responses and study of seasonal influenza now that the A(H1N1)pdm09 virus has become established as the seasonal H1N1 influenza virus.

## Introduction

Prior to 2009, pandemic plans assumed that all influenza pandemics arise from the emergence of a different antigenic subtype, as was observed for the three pandemics of the 20th Century.^1^–^3^ However, the influenza A(H1N1)pdm09 strain responsible for the 2009 pandemic arose from a sequence of reassortment events rather than antigenic shift and had a generally mild course of illness with lower than expected mortality.^4^,^5^ Nevertheless, its high transmissibility – particularly in younger age groups – and rapid global spread compared with pre-2009 seasonal influenza necessitated a pandemic response, and research studies were rapidly undertaken in various settings and populations around the globe to further characterise the clinical, virological and epidemiological features of infection.

The World Health Organization (WHO) recommends countries incorporate non-pharmaceutical interventions (such as isolation of patients and quarantine of contacts, social distancing and travel restrictions) and use of antivirals for treatment and prophylaxis into their pandemic plans to reduce transmission of pandemic influenza virus within populations.^6^,^7^ Along with understanding how and when a pandemic influenza virus is transmitted, the duration of infectiousness is a critical parameter in determining the most effective application of these mitigation measures.

The detection of virus from clinical specimens is generally equated to influenza infectiousness, with the duration dependent on several factors including age, clinical illness, treatment with antiviral agents and virus detection method.^8^,^9^ We undertook a systematic review of published literature to characterise the duration of shedding of influenza A(H1N1)pdm09 virus and identify any effects of severity of illness, age, receipt of antiviral treatment and the type of laboratory test used.

## Methods

### Search strategy and selection criteria

A literature search of the PubMed database, filtered for publication dates from 2009 onwards, was undertaken on 15 March 2013 using the keywords: H1N1[All Fields] *and* shedding[All Fields]; ‘pandemics’[MeSH Terms] *or* ‘pandemics’[All Fields] *or* ‘pandemic’[All Fields]) *and* shedding[All Fields]; shed H1N1[All Fields] *and* shed[All Fields]; (‘pandemics’[MeSH Terms] *or* ‘pandemics’[All Fields] *or* ‘pandemic’[All Fields]) *and* shed[All Fields]. Any study of humans with an outcome measure of viral shedding using any test method was eligible for inclusion in the review.

Titles and abstracts of articles returned from the searches were reviewed and were excluded from further evaluation if they: did not comprise human subjects; did not measure virus shedding; measured shedding of non-pandemic/seasonal influenza, live attenuated vaccine or oseltamivir-resistant virus only; were restricted to specialised or high-risk populations (such as patients with HIV, cancer, who were transplant recipients or otherwise immunocompromised); had five or fewer participants; or were not written in English. Shortlisted articles were then evaluated in more detail, and their reference lists searched to identify additional potentially relevant articles.

During the detailed evaluation process, studies were excluded if there were not at least three specimen collection attempts from each participant (unless a negative result or loss to follow up) in the 7 days from presentation; viral shedding was reported as mean or median virus titre, viral load or reverse-transcription polymerase chain reaction (RT-PCR) cycle threshold; or shedding duration was not reported or could not be calculated for each patient as from the day of symptom(s) onset to day of collection of the last specimen in which virus was detected. Where possible, we adjusted the data in papers that used a different definition of viral shedding duration: one day was added to the duration of viral shedding if the definition was not inclusive of the day of symptom(s) onset (e.g. defined as ‘days since’ or ‘days after’ onset); one day was subtracted from the duration of viral shedding if the definition was reported to be the day that the first negative specimen was collected *and* specimens were collected daily, otherwise the study was excluded from analysis.

Two investigators (JEF and KG) read all the articles shortlisted from the search, applied the exclusion criteria and extracted the data separately. Differences were resolved by discussion and consensus.

### Data abstraction

For each paper, we collected information on the number and age group (child or adult as defined in the manuscript, or <15 years/≥15 years respectively if not explicitly stated) of study participants, respiratory specimen sampling method and frequency, the type(s) of test used to detect influenza virus or viral RNA, the defined interval for viral shedding duration and endpoint of patient follow-up, the clinical severity (classified by the study setting: community, hospital or intensive care), antiviral treatment for study participants and – where given – those who were treated in a timely manner (generally considered to be within 48 hours of symptom(s) onset). Unless otherwise described, severity was classified as community-based illness if study participants were part of studies undertaken during the containment phase of the pandemic when many countries required isolation of patients (usually in hospitals) despite the presence of only mild illness.

We defined viral shedding duration as the number of days from day of symptom(s) onset to the day of collection of the last specimen in which influenza A(H1N1)pdm09 was detected, inclusive. Pre-symptomatic shedding and asymptomatic shedding in two studies were described separately. Summary measures of viral shedding duration (minimum, maximum, median, mean and 95% confidence interval) for each study were derived from patient record-level data, values reported in the body text, tables or survival curves. Data on the proportions of total study participants shedding virus by day of illness were extracted from tables or survival/Kaplan–Meier curves in 14 of the 22 reviewed studies. Summary measures and the proportion of participants shedding virus by day of illness were also extracted and/or calculated for the clinical severity, age group and antiviral treatment strata if the data were appropriately reported and there were six or more cases in the stratum.

### Data analysis

Meta-analyses using a random-effects model were conducted in Stata, version 10.1 (StataCorp LP, College Station, Texas, USA). Heterogeneity between studies was assessed by the *I*^2^ test, and summary estimates calculated if *I*^2^ < 80% and *P* > 0·1. To compare findings between studies, summary measures of viral shedding duration are presented in forest plots and the proportion of patients shedding virus by day of illness in survival curves. In instances where all summary measures were not reported or able to calculated from the reported data within the paper, or the definition of viral shedding duration was not given or ambiguous, the corresponding author was contacted to provide them.

## Results

A total of 214 citations were returned from the search, of which 167 were excluded following title and abstract review. Searching of article reference lists identified an additional four papers, resulting in 51 papers being evaluated in detail. A further 29 studies were excluded, mainly because of differences in the method by which virus shedding and shedding duration were measured (Table 1). A total of 22 studies were included in the review, with the number of participants in each ranging from 15 to 421. All included studies were observational in nature, with considerable heterogeneity of specimen collection method and frequency (Table 2). All studies measured viral shedding by PCR; six also measured shedding by culture. The corresponding authors of 19 studies were contacted for supplementary summary data or clarification of methodology, with responses received from nine (47%).

**Table 1:** Identified studies and reasons for exclusion

Criteria	Number of studies
Identified from search	214
Excluded after title and abstract review	167
Did not comprise human subjects	81
Did not measure virus shedding	30
Non-pandemic, vaccine or oseltamivir-resistant virus shedding	26
Restricted to specialised or high-risk populations	20
Five or fewer participants	2
Not written in English	7
Unable to be retrieved	1
Additional inclusions after search of shortlisted articles	4
Excluded after detailed evaluation	29
Shedding reported as mean virus titre/load or RT-PCR cycle threshold	10
Unable to determine patient shedding duration as onset to last positive	13
<3 specimens per patient collected and/or <7 days of follow-up	3
Study data were a subset of another included study	3
Included in the review	22

**Table 2:** Participant profiles and methodologies of studies included in the review

Study	Participants	Age groups	Clinical presentation	Treatment	Specimen type*	Sampling frequency	Test method
Beutel *et al*.^40^	25	Adults	Hospitalised (intensive care)	Oseltamivir (96%)	NPS	Twice weekly	PCR
Bhattarai *et al*.^19^	26	Children & adults	Community	Oseltamivir (12%)	NPS	Every 48 hours until 2 negative or indeterminate and negative result	PCR & culture
Cao *et al*.^41^	421	Children & adults	Community	Oseltamivir (82%)	PS or NPS	Daily until 2 consecutive negative results	PCR
Chin *et al*.^42^	15	Adults	Hospitalised	Oseltamivir (100%)	OPS	Every 2 days until 2 consecutive negative results	PCR
Cowling *et al*.^10^	45	Children & adults	Community	Strata: oseltamivir & no treatment	NTS	Three times over 7 days	PCR & culture
Duan *et al*.^43^	122	Adults	Community (quarantine & hospital observation)	Oseltamivir (100%)	PS or NPS	Daily for 7 days	PCR
Esposito *et al*.^44^	74	Children	Hospitalised	None treated	NPS	On day 3 post-onset and every 2 days until 2 negative results	PCR
Hien *et al*.^12^	292	Children & adults	Community (hospital observation)	Oseltamivir (100%)	NTS	Either daily, days 1 and 5 after admission or days 1,3 and 5 after admission	PCR
Jia *et al*.^45^	67	Adults	Community (hospital outpatients)	Chinese traditional medicine (no antivirals)	NPS	Daily for 14 days	PCR
Kay *et al*.^17^	16	Adults	Community	Oseltamivir (100%)	Nasal wash	Every Monday, Wednesday and Friday	PCR & culture
Killingley *et al*.^18^	19	Children & adults	Community & hospitalised	Strata: oseltamivir & no treatment	NS	Daily for 10 days (adults) or 14 days (children)	PCR & culture
Leung *et al*.^14^	56	Children & adults	Community (hospital observation)	Oseltamivir (96%)	NPA, NPS, NTS or TS at discretion of treating physician	At discretion of treating physician	PCR & culture
Ling *et al*.^46^	70	Adults	Community (hospital observation)	Oseltamivir (100%)	NTS	Daily until negative result	PCR
Loeb *et al*.^15^	97	Children & adults	Asymptomatic & community	Not specified	NS	Daily for 7 days then every 2 days for 3 weeks	PCR
Malato *et al*.^47^	17	Adults	Hospitalised (intensive care)	Oseltamivir (76%)	NS, broncho-alveolar lavage fluids, respiratory secretions	Not specified	PCR
Meschi *et al*.^16^	27	Adults	Hospitalised	Strata: oseltamivir & no treatment	NPS	Not specified but ≥3 per patient	PCR
Petersen *et al*.^48^	20	Adults	Hospitalised (intensive care)	Oseltamivir or zanamivir (76%)	NS or tracheal secretions	At least every 2 days	PCR
Suess *et al*.^9^	37	Children & adults	Community	Oseltamivir (35%)	Nasal wash	Daily	PCR
Suryaprasad *et al*.^13^	35	Children & adults	Community	Strata: oseltamivir & no treatment	NPS	Every 2 days until 10 days after fever cessation	PCR
To *et al*.^49^	22	Children & adults	Community (hospital observation)	Oseltamivir (95%)	NPA or NPS	Not specified	PCR
Waiboci *et al*.^50^	106	Children & adults	Community (hospital outpatients)	Oseltamivir (2%)	OP/NP specimens	Every 2 days	PCR
Xiao *et al*.^11^	156	Children & adults	Community (hospital observation)	Oseltamivir (100%)	NPS	Daily until 2 consecutive negative results	PCR

* NPS, nasopharyngeal swab; PS, pharyngeal swab; OPS, oropharyngoeal swab; NTS, nose and throat swab; NS, nasal swab; TS, throat swab; NPA, nasopharyngeal aspirate.

The mean and standard deviation of duration of viral shedding duration were available for 18 (82%) of the 22 included studies. Meta-analyses were conducted on studies grouped by the study settings of community-based cases (13 studies), hospitalised cases (three studies) and ICU cases (two studies), for which statistical heterogeneity as indicated by *I*^2^ values was 97% (*P* < 0·001), 45% (*P* = 0·165) and 86% (*P* = 0·008), respectively. Given the significant heterogeneity in most groups, the combined estimates of viral shedding duration are not reported.

### Severity of clinical presentation

**Figure 1:**
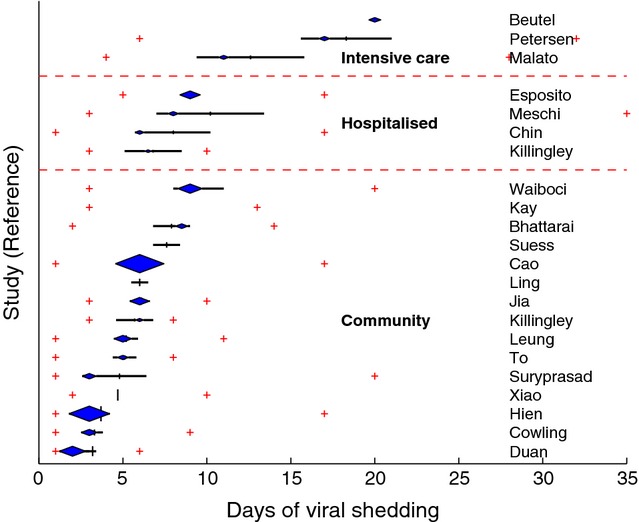
Shedding duration of influenza A(H1N1)pdm09 by study and patient setting. (Legend: cross = minimum and maximum; middle of diamond = median; area of diamond = study size; vertical line = mean; horizontal line = 95% confidence interval)

A relatively defined gradient of viral shedding duration was observed when summary measures were stratified by study setting, as a proxy for severity of clinical presentation (Figure 1). The mean duration of viral shedding was 3–9 days for community-based cases (15 studies), 7–10 days for hospitalised cases (four studies) and 13–18 days for those admitted to intensive care (three studies). The ranges of median viral shedding duration across the studies by respective settings were similar to the range of means (Figure 1). The studies involving those hospitalised and admitted to intensive care had relatively wide 95% confidence intervals, with generally smaller study sizes and a wider range of shedding duration. Shedding duration was longer for a higher proportion of hospitalised cases and longer still among cases in intensive care, with 80% or more cases still shedding virus at 18 days in two of the three studies (see survival curves in Supplementary Data). The maximum shedding duration in these studies was 28, 32 and 158 days. Between 71% and 86% of patients in the three studies of intensive care patients had one or more risk factors for severe influenza such as pregnancy, obesity, cardiovascular disease, diabetes mellitus, immunosuppressive therapy or chronic pulmonary, renal or liver disease.

### Age

**Figure 2:**
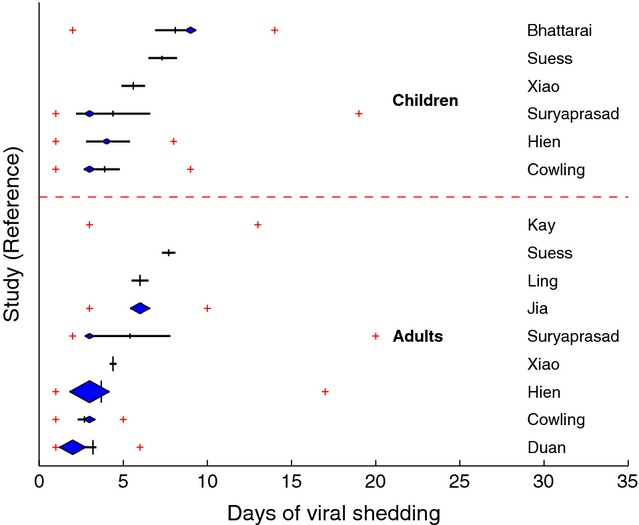
Shedding duration of influenza A(H1N1)pdm09 in studies of community-based cases, by study and age group.

Given the small number of studies among hospitalised and intensive care patients, age stratification was restricted to studies of community-based cases. Summary measures of viral shedding duration were available for 15 adult or children strata from ten studies. There was little difference in the ranges of mean viral shedding duration between the adults (3–8 days) and children (4–8 days) with similar observations for the respective median values (Figure 2). Comparison of viral shedding duration measured by PCR between community-based child and adult cases was made directly in five studies; children had longer shedding duration in three of the studies, two by a mean of 1·2 days^10^,^11^ (of which *P* < 0·01 for one of the studies)^11^ and the other by 0·4 day^12^ but was longer in adults in the other studies by 0·4^9^ and 1·0 days.^13^ An additional paper that compared shedding duration in community-based cases but measured by viral culture found a mean of 5·7 days in children compared with 3·7 days in adults (*P* = 0·03).^14^

### Asymptomatic shedding

One study by Loeb *et al*.,^15^ conducted over several influenza seasons among a cohort of relatively isolated communal farming communities, measured shedding duration for cases of asymptomatic influenza A(H1N1)pdm09. Of the 97 participants in the study, 12 (12%) were asymptomatic and had a mean viral shedding duration of 3·2 days (95% CI: 2·0–4·4) compared with 4·8 days (95% CI: 4·2–5·4) for all participants. Only one other study by Suess *et al*.^9^ described asymptomatic cases. Surveillance of 30 laboratory-confirmed index cases identified 15 secondary cases, of which three (20%) were asymptomatic, although no data on shedding duration were available. The study by Loeb *et al*. was also the only one included in the review to systematically assess pre-symptomatic shedding and compare shedding duration of influenza A(H1N1)pdm09 with pre-2009 seasonal influenza over a 2-year study period. The study found that nine (11%) of 85 symptomatic cases shed virus in the day before acute respiratory illness onset and three (4%) up to 3 days before onset and that with a mean shedding duration of 4·8 days, influenza A(H1N1)pdm09 was comparable to seasonal H1N1 and type B influenza (5·2 and 4·9 days respectively) but longer than seasonal H1N1 (3·4 days, *P* = 0·03).^15^

### Antiviral treatment

**Figure 3:**
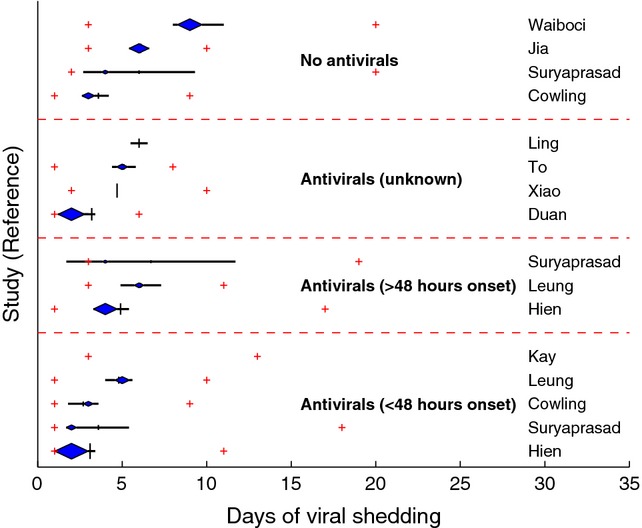
Shedding duration of influenza A(H1N1)pdm09 in studies of community-based cases, by study and antiviral treatment.

Summary measures of viral shedding duration stratified by treatment modality were available from 11 studies of community-based cases, of which four further differentiated by whether or not oseltamivir was administered within 48 hours of illness onset. The range of mean values for viral shedding duration in studies of those treated with oseltamivir within 48 hours of illness onset (3–5 days) was lower than those for which treatment was administered after 48 hours of onset (5–7 days) and for those not treated (4–9 days) (Figure 3). Similar results were observed for median values of shedding duration (Figure 3). Several studies directly compared treatment modalities. Hien *et al*.^12^ observed statistically significant shorter shedding duration among those treated within 48 hours of onset compared with those treated after 48 hours; this observation was also made by Leung *et al*.,^14^ but the difference was only statistically significant when shedding was measured by viral culture rather than RT-PCR. Similarly, shorter shedding duration was found by Cowling *et al*. and Suryaprasad *et al*. in those treated within 48 hours of illness onset compared with treatment after 48 hours or no treatment, but the difference was not significant.^10^,^13^ In contrast, a study of hospitalised cases by Meschi *et al*.^16^ noted a shorter, but not statistically significant, shedding duration in untreated cases compared with those who received oseltamivir.

### Culture versus RT-PCR

In addition to RT-PCR, five studies measured viral shedding by culture. Two studies measured viral shedding by culture for all study participants,^14^,^17^ and for 63%^18^ and 73%^10^,^19^ of patients in the other three studies. With the exception of one study in which median values were the same and the means differed by 0·3 day^10^, the mean and median durations of viral shedding were 1·5–2 days shorter when measured by culture. The maximum shedding duration was shorter by 2–3 days in four studies^10^,^14^,^17^,^18^ and 6 days in the other.^19^

## Discussion

Several studies have reported that duration of pre-2009 seasonal influenza virus shedding is longer in children^20^–^22^ and has become a widely accepted assumption in text books^23^ and pandemic planning documents.^6^ However, we did not demonstrate longer shedding duration of influenza A (H1N1)pdm09 among children compared with adults, either between or within studies. Three of the five studies in the review that directly compared shedding duration in adults to children observed shedding to be longer in children, whilst three other studies not included in the review – primarily because shedding was measured as virus titre or load – were also split in their findings: two studies found significantly longer shedding duration in children^24^,^25^, whilst no difference was found in another.^26^ A further two studies reported no difference in the proportion of adults and children with prolonged viral shedding of more than 7 days.^27^,^28^ If not related to statistical anomalies, the absence of a difference in influenza A(H1N1)pdm09 shedding duration between children and adults may in part be explained by their similar susceptibility to the then-novel pandemic strain,^29^ as opposed to pre-2009 seasonal influenza in which adults have more previous exposures and greater cross-protective immunity. However, there are few papers comparing viral shedding across several years to compare shedding in the pandemic and seasonal strains to support this hypothesis; whilst one study found a significantly longer duration of pandemic virus shedding compared with H3N2,^15^ another found little difference.^10^

As to be expected, progressively longer shedding duration cases of influenza A(H1N1)pdm09 infection were observed when studies were stratified into community (mean and median range: 2–9 days), hospital (6–10 days) and intensive care (13–20 days) settings. Prolonged shedding of more than 14 days was still seen for a small proportion (less than 20%) of patients in several of the community-based studies, but is not unexpected given that prolonged shedding can occur even in immunocompetent patients with non-mutated virus.^30^ With 70% or more of the cases in the three studies in ICU settings reported to have one or more risk factors for severe infection, the higher median values for duration of infection (11–20 days) and an upper range of 158 days are consistent with studies restricted to immunocompromised patients.^31^–^33^ The observation of generally shorter viral shedding duration in studies where cases received oseltamivir treatment within 48 hours of illness onset was consistent with the literature,^34^ despite relatively few strata for comparison. However, the author of one hospital-based study in which longer shedding was observed in treated patients compared with untreated patients^16^ indicated by correspondence that this was probably a consequence of the treated group including patients with a more severe clinical presentation, suggesting that at least in some instances, differential inclination to treat can influence reported viral shedding duration.

The biggest challenge in extracting and compiling individual study data for this review was the variation in definitions, where provided, of the primary outcome measure of duration of viral shedding. The variability applied to the start point of shedding duration (either the day of symptom onset, first positive test or treatment initiation), the endpoint (either the day of the last positive or first negative test) and how days of shedding duration were calculated (either by counting the starting point day as one day of viral shedding, or using the days difference between the start and endpoints). The latter component of shedding duration was particularly poorly defined in many studies and in the absence of confirmation from corresponding authors needed to be assumed based on table, figure or axis titles, or descriptions in the main text. Using the day of the last positive result as the viral shedding duration endpoint is an additional limitation because it will underestimate viral shedding duration in studies where patients are not sampled every day. Kay *et al*.^17^ used statistical modelling to account for the gap between last positive and first of two consecutive negatives as the endpoint of viral shedding. Loss of study participants to follow up, an inevitability particularly during the early stages of a pandemic, will also underestimate viral shedding duration. Furthermore, it cannot be assumed that patients are shedding the same quantity of virus throughout the course of their illness (as demonstrated by shedding studies measuring viral load,^10^,^26^,^35^,^36^ most of which were outside the scope of this review) or indeed continually shed virus throughout the course of their infection. More than half of the reviewed studies attempted to avoid underestimation of viral shedding duration caused by intermittent shedding by requiring at least two consecutive negative specimens as an endpoint of testing follow-up, which is shown schematically for several cases in three of the reviewed studies.^12^,^13^,^19^ Whilst a standardised measure of viral shedding duration was able to be applied to 22 studies in this review, numerous adjustments and assumptions were needed, and a further 13 had to be excluded. The development and adoption of standard parameters, which we have proposed in Box 1, would assist in simple and rapid assessment and comparison of influenza viral shedding duration that could reliably inform mathematical modelling (for which small variations in viral shedding duration, as a proxy for the period of infectiousness, are very sensitive) and exclusion policies, particularly during the early stages of a pandemic.

**Box 1 Proposed standard parameters for measurement and reporting of influenza viral shedding duration**

- Unless measuring pre-symptomatic or asymptomatic shedding, the duration of viral shedding should be defined as from the day of symptom(s) onset to the day on which the last positive specimen was collected.
- Counting of the number of days of viral shedding duration should be inclusive of (rather than the difference between) the day of symptom(s) onset and the day on which last positive specimen was collected.
- Specimen collection should continue until two consecutively collected specimens both test negative.
- Where administratively possible, specimens should be collected daily but not less than one every 2 days.
- The age threshold for classification as a child or adult should be clearly defined.
- Record the date (or day with respect to symptom onset) of the commencement of antiviral therapy, or that no antiviral therapy was administered.

Additional methodological heterogeneity between studies also limits the scope of the review findings and precluded meta-analysis. Eleven different specimen types were collected with varying frequency in the 22 studies included in the review and likely have varying sensitivities, particularly during the later stages of infection. Supporting this are two studies that showed higher influenza A(H1N1)pdm09 viral loads^37^ and sensitivity^9^,^38^ of RT-PCR testing of nasopharyngeal aspirate and nasal wash specimens compared with nasopharyngeal and nose/throat swabs. Detection of virus by RT-PCR is a more sensitive method than viral culture, and this was shown by Cheng *et al*.^38^ for influenza A(H1N1)pdm09 and reflected in the relative measures of viral shedding duration in the four studies in the review that compared the two methods. An advantage of viral culture is that it provides a measure of viable/infectious virus, whereas PCR may also detect non-viable viral RNA; however, the extent to which detection of non-viable RNA contributes to measures of viral shedding duration is unclear. Studies included in the review also differed by the age at which participants were classified as children, varying from 12 years or less to 15 years or less. However, given little difference in viral shedding duration was observed between children and adults in general, the impact of this variation in definitions is likely to be neutral. A further limitation of the review is that there was little insight into pre-symptomatic and asymptomatic shedding; only one study examined these but given its setting in isolated communal farming communities in Canada is unlikely to be representative.^15^ One study that studied shedding in household contacts of index cases but was excluded from the review because viral shedding was reported as median viral load showed asymptomatic shedding in 12% and pre-symptomatic shedding up to 4 days prior to symptom onset in one (4%) of 28 secondary cases.^26^

This review has provided insights into viral shedding duration of influenza A(H1N1)pdm09 and the relative effects of age, clinical severity and oseltamivir treatment. Additional reviews examining viral loads and correlation of symptoms over time may provide further insights into the relative infectivity and transmissibility of influenza A(H1N1)pdm09 and are warranted now that influenza A(H1N1)pdm09 has become established as the seasonal H1N1 influenza virus and that there is a large body of literature examining its properties. Understanding the infectivity of emerging novel influenza strains by synthesis of the wide array of research studies could be greatly enhanced by a standardised approach to measurement of viral shedding, and such guidelines would be a useful addition to global research planning documents such as the ‘WHO Public Health Research Agenda for Influenza’.^39^

## Supporting Information

**Figure S1** Proportion of community setting study cases positive for influenza A(H1N1)pdm09 by day of virus shedding and oseltamivir treatment. **Figure S2** Proportion of hospital setting study cases positive for influenza A(H1N1)pdm09 by day of virus shedding and oseltamivir treatment. **Figure S3** Proportion of ICU setting study cases positive for influenza A(H1N1)pdm09 by day of virus shedding and oseltamivir treatment.
