# Supplementary material for: Systematic review of influenza A(H1N1)pdm09 virus shedding: duration is affected by severity, but not age
Source: Influenza Other Respir Viruses. 2013 Dec 2;8(2):142–50. doi: 10.1111/irv.12216 (PMC4186461; doi:10.1111/irv.12216)
Supplement: Supplementary file 1 [file irv0008-0142-SD1.doc]

Appendix Figure 1: Proportion of community setting study cases positive for influenza A(H1N1)pdm09 by day of virus shedding and oseltamivir treatment

(Legend: solid line = treated with 48 hours of onset; dashed line: treated after 48 hours of illness onset; dashed and dotted line: treatment timing unspecified; dotted line: no treatment)


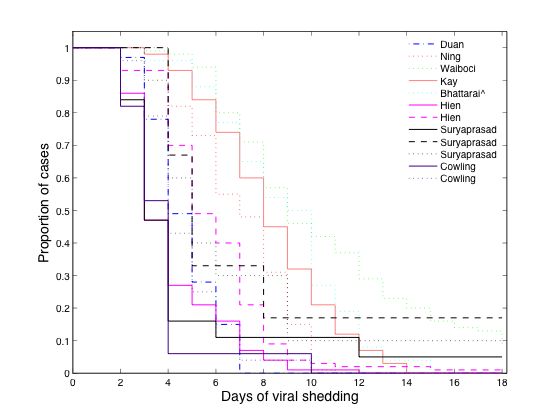


^ 12% received oseltamivir

Appendix Figure 2: Proportion of hospital setting study cases positive for influenza A(H1N1)pdm09 by day of virus shedding and oseltamivir treatment


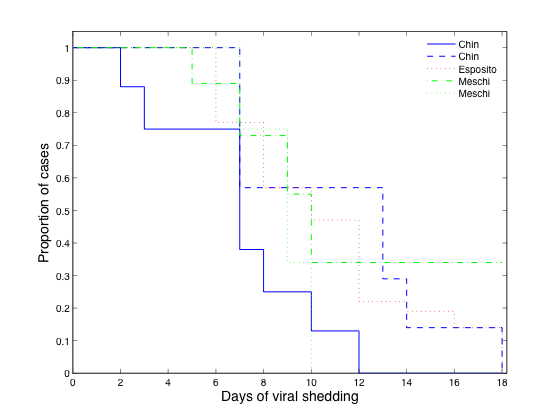


Appendix Figure 3: Proportion of ICU setting study cases positive for influenza A(H1N1)pdm09 by day of virus shedding and oseltamivir treatment


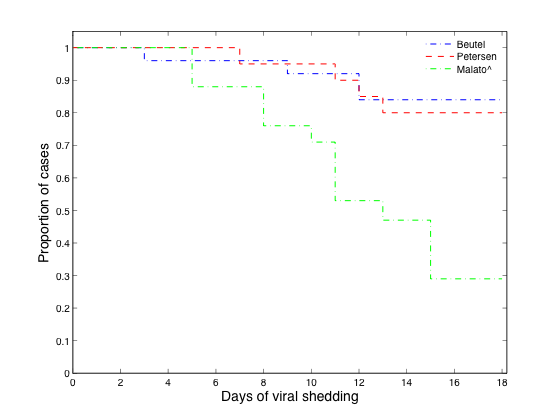


^ 76% received oseltamivir
